# Qualitative exploration of awareness about risk factors and transformative behaviours among Pakistani patients with coronary artery disease

**DOI:** 10.1136/bmjopen-2024-084566

**Published:** 2024-11-24

**Authors:** Rubina Barolia, Adnan Yaqoob, Laila Ladak, Aamir Hameed Khan, Furqan Yaqub Pannu

**Affiliations:** 1 School of Nursing and Midwifery, Aga Khan University, Karachi, Pakistan; 2 Nursing Education, Shaukat Khanum Memorial Cancer Hospital and Research Centre, Lahore, Pakistan; 3 Department of Cardiology, Ziauddin Medical University, Karachi, Sindh, Pakistan; 4 Department of Cardiology, Mayo Hospital, Lahore, Pakistan

**Keywords:** Coronary heart disease, Health Literacy, Cardiovascular Disease

## Abstract

**Abstract:**

**Objectives:**

To explore patients’ awareness of risk factors for coronary artery disease (CAD) and experiences of the behavioural modifications, they made during their recovery through in-depth semistructured interviews.

**Design:**

Qualitative exploration was undertaken as part of a larger project to answer the study questions.

**Setting:**

Participants were recruited from a public-sector tertiary care hospital in Lahore Pakistan.

**Participants:**

A total of 20 participants, men and women, who had more than 6 months of experience living with CAD, were recruited using a purposive sampling technique.

**Results:**

The data were organised and analysed using NVIVO software. An inductive coding approach was used to generate codes, categories and themes. Three themes emerged from participants’ data: lifestyle choices and behavioural factors, reflective awareness and lifestyle changes, holistic perspectives and health management.

**Conclusions:**

The present study’s findings align with recent literature, emphasising the complex nature of risk factors contributing to heart disease. The participants’ understanding and behavioural changes show the importance of health awareness and preventive measures in reducing cardiovascular risks.

**STRENGTHS AND LIMITATIONS OF THIS STUDY**

- Qualitative data were obtained from patients who had suffered a heart attack.
- The qualitative nature of the data provides a holistic view by exploring patients’ awareness and lifestyle modifications within their natural setting, which helps to understand the context.
- An authentic tool was used for data analysis (NVIVO).
- Results are typically specific to participants who suffered heart attacks, which makes the findings difficult to generalise on other cardiac populations.
- The data collected are based on self-reports from participants, which may be subject to recall bias.

## Introduction

 Understanding heart attacks is crucial for patients as it empowers them to make informed decisions about their lifestyle. A body of literature supports the significance of patients’ comprehension of heart attacks for better health outcomes. Furthermore, patient education improves medication adherence, and lifestyle modifications and reduces risk factors associated with recurrent cardiac events.^1 2^ Additionally, studies highlight that informed patients are more likely to seek timely medical attention during the onset of symptoms, leading to better outcomes and reduced morbidity and mortality.^3^ Patient understanding also plays a pivotal role in successfully implementing secondary prevention strategies, such as dietary changes, regular exercise and smoking cessation.^4^ The literature emphasises the importance of patients’ comprehension of coronary artery disease (CAD) for improved self-management, treatment plan adherence and overall cardiovascular outcomes.

The literature consistently supports that patients’ understanding of CAD risk factors, symptoms and preventive measures contributes to better adherence to medical recommendations and positively influences psychosocial health. This emphasises the need to explore Pakistani patients’ perceived risk factors and lifestyle modifications they have made to manage cardiovascular health. By exploring participants’ experiences and attitudes, the research seeks to uncover the depth of awareness regarding the connection between lifestyle and cardiovascular health.

### Research questions

1. What is the awareness of Pakistani patients about the risk factors of CAD?
2. What lifestyle modifications do Pakistani patients make during their recovery from CAD?

### Research objectives

1. To explore Pakistani patients’ awareness of risk factors for CAD.
2. To explore Pakistani patients’ experiences of the behavioural modifications they make during their recovery from CAD.

### Significance

The research identifies participants with a proactive approach to health, particularly those with coronary angioplasty. Understanding their belief and behaviours provides insights into potential preventive measures and overall health strategies. Additionally, findings provide a rich and careful understanding of patients’ perceptions, experiences and behaviours related to heart disease. The findings may also inform targeted interventions, educational programmes, and healthcare policies to reduce the incidence of heart-related issues by addressing the identified risk factors. Furthermore, the current study’s findings provide valuable insights into participants’ understanding of diverse risk factors contributing to heart disease, emphasising the importance of health awareness and paving the foundation for developing contextual interventions such as home-based cardiac rehabilitation in reducing cardiovascular risks and improving overall health.

## Methods

The qualitative data was collected as part of a larger project. A qualitative exploration of any phenomenon helps the researcher get an in-depth understanding of multiple realities. This research focused on the interpretivist research paradigm to explore and understand the patients’ perspectives. The data were collected from a public tertiary care hospital. The public sector tertiary care hospital was selected to enrol patients from diverse socioeconomic backgrounds to get a clearer image of real experience after a heart attack.

### Sampling strategy

Participants of both genders (men and women) who have been dealing with heart attacks for at least the last 6 months were recruited through a purposive sampling technique. The reason for employing a purposive sampling technique was to get those participants involved who met the inclusion criteria and could give in-depth data.

### Data collection methods

Patients who met the selection criteria were approached from the outpatient department of a public tertiary care hospital. After obtaining the written informed consent, participants were given the choice to do it physically in the hospital’s dedicated room or telephonically at home at their convenience. The data were collected by the researcher through in-depth semistructured interviews. The interview duration ranged from 40 min to 50 min. A total of 20 participants were interviewed based on data saturation. Data saturation in this study was achieved when no new themes emerged from the participants’ data.

### Questionnaire guide

An interview guide was developed using the research team’s experience and help from the literature. Open-ended questions were converted into the national language and asked by the patients for ease of understanding. Before starting the final interviews with the research participants, the pilot qualitative interviews were conducted with heart patients to see the clarity and understandability of questions by patients. Patients included in the pilot interviews were excluded from the primary sample size. After the pilot interviews, a few questions were modified based on the responses received, and final interviews were conducted until data saturation.

### Data processing and analysis

Two independent transcribers transcribed the recorded interviews into the original language. During the transcription, the expressions of participants and notes taken during the interviews were also included in the text. Any identifiable information about the participants in the recordings was omitted and did not make a part of the transcriptions. The transcriptions from both transcribers were evaluated for any discrepancy. Then, the transcriptions were translated into English, and integrity was maintained by asking participants if there were any changes to their responses. The transcription remained anonymous and coded with a unique number. Furthermore, each transcription was added to the NVIVO software for line-by-line coding. Similar coding extracted from all the transcriptions was merged into groups to make subthemes, whereas similar groups/subthemes were again merged to create themes.

### Techniques to enhance trustworthiness

The credibility of qualitative findings was ensured by member checking to ensure the data accuracy in the interview transcripts. The participants were contacted back if there was any ambiguity in understanding the responses, and after the confirmation of responses, the transcript was added to the software for analysis.

## Results

Out of 20 participants, 14 were men and 6 were women. The mean age of participants was 51.9 years. 11 men were still smokers, 8 were treated conservatively, whereas 12 underwent percutaneous coronary intervention. Participants’ data revealed an understanding among participants regarding the heart attack and perceived risk factors. Three themes emerged from participants’ data: lifestyle choices and behavioural factors, reflective awareness and lifestyle changes, holistic perspectives and health management (table 1). The themes encompass dietary habits, lifestyle choices, family history and a multifaceted approach to preventive measures, highlighting the importance of holistic health awareness in mitigating heart-related risks.

**Table 1:** Themes and quotations

Themes	Quotes	Participants
Lifestyle choices and behavioural factors	*I think an unhealthy diet is a cause, such as a diet with high fat and meat daily, is very dangerous for the heart. I used to eat such and liked to add extra salt while eating…this should be avoided:*	(*P17, M, late 60s*)
	*I am fond of cold drinks; I drink a lot of Coca-Cola and Pepsi…they may affect heart…I do not think so because these drinks are now normally served to almost every guest…*	(*P15, F, early 50s*)
	*I remain worried about my cholesterol; it never came down…I used to eat red meat, and due to this, I developed high blood pressure…and, after a few days, a heart attack. I do not take such foods, which has hurt me greatly.*	(*P20, M, late 50s*)
	*I am a smoker, and now I realize that it could cause heart disease.*	(*P17, M, mid 60s*)
	*I realize that smoking has caused me this disease…*	(*P2, M, late 60s*)
	*I realize I used to eat unhealthy food…I am a smoker, but I smoke very little, so I do not think very little smoking can cause heart effects.*	(*P7, M, early 40s*)
	*See…except smoking. I did not take it, which could be harmful because I used to eat vegetables more in my diet than meat or chicken… I think smoking is the cause of my condition. I quit it now and feel much better without having shortness of breath (smiles)…*	(*P6, M, early 70s*)
	*I heard that it affects lungs but never heard of its effects on the heart…even if it has, I do not smoke.*	(*P12, F, mid 40s*)
	*I have sugar, and due to this, I had a heart attack because I read somewhere that sugar damages veins, and doctors told me that those who do not care get heart attacks again and again.*	(*P19, F, late 50s*)
	*I now realize that it is smoking and diabetes which led me to develop heart disease…I never believed, but now I realized…*	(*P9, M, mid 50s*)
	*It is due to family history, and I am obese, a smoker, and diabetic. These all I think had caused heart disease, isn’t it?*	(*P11, M, early 50s*)
Reflective awareness and lifestyle changes	*I understand that what we eat and do with ourselves deeply affects our health. I was a chain smoker for the past 40 years, and I never paid attention to its effects on my health despite multiple counseling by my family and friends, but now…I had hypertension, and I did not take it seriously… I realize that smoking has caused me this disease…*	(*P5, M, late 60s*)
	*I was looking back at my life and asking myself questions about what I did wrong that led me to where I am today. I recall that I smoked so much.*	(*P8, M, late 50s*)
	*I think smoking is the cause of my condition. I quit it now and feel much better without having shortness of breath (smiles)…*	(*P6, M, late 70s*)
	*I eat chhalia (betel nuts), smokeless tobacco (talking about pan and gutka), and drink much caffeine…I think that has caused me heart disease…*	(*P13, M, early 40s*)
	*I eat chhalia but I do not believe it has any relationship with the heart disease…*	(*P16, F, early 40s*)
Holistic perspectives and health management	*I thought at that time that it is first and last if I do not care for myself this time…because if I get the second, I will be over…I had 2–3 coronary angioplasty in my heart…there is no chance for the next…so I teach myself to remain active and avoid things that cause heart attack…*	(*P20, M, mid 50s*)
	*I think those who remain inactive get heart disease…*	(*P18, M, late 50s*)
	*I think heart disease runs in the family…my mother and father have this disease, so I have…people kept on searching for the cause, but they need to understand that it is from almighty ALLAH…*	(*P5, M, late 60s*)
	*I have no idea what is the reason for my disease…I believe it is from Almighty…*	(*P19, F, early 50s*)
	*I had hypertension, and I did not take it seriously…I kept on smoking and used to eat fatty foods…I realize that smoking has caused me this disease…*	(*P4, M, early 70s*)
	*Before my heart attack, I used to take extra pills for my blood pressure, which used to increase frequently. I take two different blood pressure medications now: a 10 mg one that I take twice daily and a 5 mg one that I take only once. It is not only BP, but the sugar also disturbed me a lot…I do not take too much sugar but a very little occasionally.*	(*P6, M, early 70*)
	*Heart patients need to know what normal BP and sugar are because if they know they can manage it easily and if they ignore, they invite trouble for themselves.*	(*P11, M, early 50s*)
	*I realized that my behavior was not good at that time towards my health, but now I have changed it significantly…*	(*P8, M, early 50s*)

### Theme 1: lifestyle choices and behavioural factors

Lifestyle choices are pivotal in shaping cardiovascular health, as evidenced by the participants’ histories. A total of four subthemes have emerged that emphasise the intricate relationship between lifestyle choices, including dietary patterns, cholesterol management and the awareness of smoking and its connection to diabetes, in influencing cardiovascular health:

The recognition of dietary habits as a significant risk factor for heart disease reflects a critical awareness of the impact of nutrition on cardiovascular health. Participants expressed concern about cholesterol levels, emphasising the association between dietary choices, such as consuming red meat, and the development of high blood pressure. This awareness stresses the importance of managing individual health signs to lessen heart-related risks. Moreover, the recent realisation among participants regarding the adverse effects of smoking on the heart demonstrates a critical reflection on past habits. Additionally, the acknowledgement of the combined impact of smoking and diabetes on heart disease highlights a deeper understanding of the synergistic effects of multiple risk factors.

#### Subtheme 1.1: unhealthy diet

The participants shared their beliefs that an unhealthy diet, particularly one rich in high-fat and daily meat consumption, carries a significant risk to heart. The comment about adding extra salt to meals highlights awareness of the potential dangers associated with excessive sodium intake. The participant is committed to avoiding such dietary habits, signalling a proactive approach towards health promotion, as he shared:

I think an unhealthy diet is a cause, such as a diet with high fat and meat daily, is very dangerous for the heart. I used to eat such and liked to add extra salt while eating…this should be avoided: (P17, M, late 60s)

Additionally, another participant shared a different contextual perspective that many food choices are unhealthy but adopted by society as a culture and norm. She shared that she drinks a lot of carbonated drinks routinely and these are also served to the guests as a norm in society; furthermore, the response also showed a lack of awareness about the potential harms of soft drinks on cardiac health:

I am fond of cold drinks; I drink a lot of Coca-Cola and Pepsi…they may affect heart…I do not think so because these drinks are now normally served to almost every guest… (P15, F, Early 50s)

#### Subtheme 1.2: cholesterol concerns

The participant’s response conveys ongoing concern about elevated cholesterol levels, attributing it to past habits of consuming red meat. The association between high blood pressure development and subsequent heart attack after engaging in such dietary practices is demonstrated by the participant’s response. The participant conveys a sense of regret and emotional distress, emphasising the profound impact of unhealthy eating habits on their health. The decision to withdraw such foods appears to be a significant lifestyle change, potentially driven by the personal experience of adverse health outcomes. This description emphasises the clear connection between dietary choices, cholesterol management and cardiovascular events in the participant’s life, as he shared:

I remain worried about my cholesterol; it never came down…I used to eat red meat, and due to this, I developed high blood pressure*…*and, after a few days, a heart attack. I do not take such foods, which has hurt me greatly. (P20, M, late 50s)

Furthermore, another participant’s response reveals a dual risk factor scenario involving both smoking and a diet high in fatty foods. The acknowledgement of these concurrent habits suggests an understanding of the combined effects on cardiovascular health. The participant’s statement, *I kept on smoking and used to eat fatty foods (P4, M, 60s),* implies a past perseverance in these behaviours. While the response does not provide clear details on current actions, it does suggest an awareness of the potential harm associated with the combination of smoking and a high-fat diet. This response may indicate a crucial moment of reflection or recognition, potentially paving the way for lifestyle changes to reduce cardiovascular risk factors.

#### Subtheme 1.3: smoking realisation

Similarly, the responses from patients provide valuable insights into their awareness and perceptions regarding lifestyle factors and their impact on health. The response indicates a shift in awareness, with the participant acknowledging the potential link between smoking and heart disease. The response suggests a realisation that was previously lacking:

I am a smoker, and now I realize that it could cause heart disease. (P17, M, mid 60s)

The respondent goes a step further by directly attributing the disease to smoking, indicating a sharp awareness of the consequences of this habit. This recognition could potentially catalyse behavioural change:

I realize that smoking has caused me this disease… (P2, M, late 60s)

Furthermore, the response also reflects a perspective acknowledging unhealthy dietary habits while diminishing the impact of limited smoking on health. It suggests a need for further education on the cumulative effects of various lifestyle factors:

I realize I used to eat unhealthy food…I am a smoker, but I smoke very little, so I do not think very little smoking can cause heart effects. (P7, M, early 40s)

On the other hand, a participant shared a more comprehensive understanding of the role of diverse lifestyle choices, such as diet and smoking. The participant recognises the harm caused by smoking, takes proactive steps by quitting and reports improved health, particularly regarding respiratory health. It indicates a positive behavioural change and emphasises the potential benefits of lifestyle modifications:

See…except smoking. I did not take it, which could be harmful because I used to eat vegetables more in my diet than meat or chicken… I think smoking is the cause of my condition. I quit it now and feel much better without having shortness of breath (smiles)… (P6, M, early 70s)

Additionally, a participant who was a non-smoker shared her awareness about the effects of smoking on the lungs; however, she showed a lack of awareness about its effects on the heart. Despite several educational campaigns on the effects of smoking and its proven facts that it affects the heart, several heart attack survivors are still unaware of it. Even if they are not smoking, they should know the effects of passive smoking and its effects. Furthermore, the response indicates that being a non-smoker, it gave her a sigh of relief that at least she does not smoke

I heard that it affects lungs but never heard of its effects on the heart…even if it has, I do not smoke. (P12, F, Mid 40s)

#### Subtheme 1.4: top of form smoking and diabetes connection

It is interesting to note that the patient’s response reflects their perceived connection between diabetes and heart attack. The concern about vascular health and the impact of sugar on veins is valid and highlights the importance of diabetes management in preventing cardiovascular disease. The mention of doctors warning about recurring heart attacks for those neglecting care highlights the seriousness of the patient’s condition and the need for effective management. The patient’s sense of personal responsibility tied to diabetes management and its impact on cardiovascular health is commendable and demonstrates the importance of patient education and self-care in minimising the risks associated with heart disease.

I have sugar, and due to this, I had a heart attack because I read somewhere that sugar damages veins, and doctors told me that those who do not care get heart attacks again and again. (P19, F, late 50s)

Similarly, it is encouraging to note that the patient in question has gained a better understanding of the risk factors associated with heart disease, such as smoking and diabetes. Their admission of initial disbelief and subsequent recognition of the connections between lifestyle factors and their cardiac condition highlights the importance of raising awareness about the impact of lifestyle choices on heart health. This transformative shift in the patient’s awareness is significant and reinforces the need for continued education on preventive measures for heart disease.

I now realize that it is smoking and diabetes which led me to develop heart disease…I never believed, but now I realized… (P9, M, mid 50s)

Furthermore, it is noteworthy that the patient attributes their heart disease to a combination of family history, obesity, smoking and diabetes. This response reflects a multifactorial perspective on the origins of their condition, recognising the interplay of genetic predisposition and modifiable risk factors. The patient’s query, ‘These all I think had caused heart disease, isn’t it?’ indicates a desire for confirmation and an understanding of the various factors that contribute to their cardiovascular health. These responses stress the complex interrelationships between lifestyle choices, health conditions and individual perceptions in shaping patients’ understanding of their heart disease. It highlights the need for a patient-centred approach that considers each individual’s unique circumstances and perspectives in designing effective prevention and treatment strategies.

It is due to family history, and I am obese, a smoker, and diabetic. These all I think had caused heart disease, isn’t it? (P11, M, early 50s)

### Theme 2: reflective awareness and lifestyle changes

Reflective awareness and lifestyle changes are pivotal in the participants’ journey towards better health. Reflecting on past habits is evident as patients retrospectively evaluate their lifestyles, with a particular emphasis on aspects such as excessive smoking. This self-examination shows a cognitive shift, emphasising a better awareness of the impact of past behaviours on their cardiovascular health. Recognition of unhealthy habits prompts a lifestyle change as participants commit to healthier choices, including smoking cessation. Moreover, the acknowledgement of the role of substances like chhalia, smokeless tobacco and caffeine in contributing to heart disease showcases a broader understanding of risk factors beyond conventional ones. This comprehensive awareness fosters personal growth and serves as a foundation for proactive lifestyle changes, ultimately contributing to improved heart health outcomes.

#### Subtheme 2.1: reflecting on past habits

The responses by participants reflect an acknowledgement of lifestyle choices’ profound impact on health. The patient recognises the connection between prolonged chain-smoking behaviour over the past 40 years and the development of hypertension. Despite previous counselling from family and friends, there was a lack of awareness or acknowledgement of the health consequences during the years of smoking. The participant’s statement suggests a transformative realisation, wherein the participant now understands the direct link between their smoking habit and the onset of hypertension. This shift in awareness indicates a critical moment of self-reflection and a recent understanding of personal responsibility for health:

I understand that what we eat and do with ourselves deeply affects our health. I was a chain smoker for the past 40 years, and I never paid attention to its effects on my health despite multiple counseling by my family and friends, but now…I had hypertension, and I did not take it seriously… I realize that smoking has caused me this disease… (P5,M, late 60s)

The response further highlights the retrospective contemplation of life choices and their consequences. The individual expresses a sense of regret and self-inquiry, identifying a history of heavy smoking as a factor contributing to their current health condition. The narrative suggests a desire for self-awareness and a willingness to connect the dots between lifestyle choices and health outcomes. It highlights the significance of reflection and realisation in the context of health behaviours, illustrating a shift in perspective towards a more informed and accountable approach to personal health:

I was looking back at my life and asking myself questions about what I did wrong that led me to where I am today. I recall that I smoked so much. (P8, M, late 50s)

#### Subtheme 2.2: change in lifestyle

Participants express a positive change in lifestyle, including quitting smoking. The reported improvement in health, indicated by reduced shortness of breath, reflects the potential benefits of adopting a healthier lifestyle. The patient’s response suggests a notable awareness of the potential link between smoking and current health conditions. By acknowledging that smoking is the cause of a heart attack, the patient demonstrates a clear understanding of the impact of this behaviour on respiratory health. The decision to quit smoking is a positive step towards improving health. The mention of feeling ‘much better’ without experiencing shortness of breath indicates an immediate positive change in their health since quitting smoking. Including a smile further shows a sense of accomplishment and satisfaction with the decision to stop smoking. This response reflects personal intervention in addressing health issues and implies a positive trajectory in the patient’s overall health and quality of life after making this lifestyle change. It highlights the importance of smoking cessation as a beneficial intervention for respiratory and cardiovascular health:

I think smoking is the cause of my condition. I quit it now and feel much better without having shortness of breath (smiles)… (P6, M, late 70s)

#### Subtheme 2.3: other substances and heart disease

The response from the patient sheds light on certain lifestyle habits that may be perceived as potential contributors to their heart disease. The mention of consuming chhalia (betel nuts), smokeless tobacco (referring to pan and gutka) and a high intake of caffeine suggest a complex interplay of multiple risk factors. Betel nuts are known to contain arecoline, which may have cardiovascular effects. Additionally, excessive caffeine consumption has been linked to elevated blood pressure and increased heart rate.

The patient’s belief that these habits have caused their heart disease reflects a certain level of health awareness and personal attribution to lifestyle choices. From a healthcare perspective, it underscores the importance of addressing and modifying these risk behaviours as part of a comprehensive cardiovascular disease management strategy. The patient’s acknowledgement of the potential connection between their habits and heart disease could serve as a valuable starting point for health education and behaviour change interventions, emphasising the significance of adopting healthier lifestyle practices to mitigate cardiovascular risks. Overall, the response underscores the complex relationship between lifestyle choices and health outcomes, emphasising the need for tailored interventions that address the specific habits mentioned by the patient.

I eat chhalia (betel nuts), smokeless tobacco (talking about pan and gutka), and drink much caffeine…I think that has caused me heart disease… (P13, M, early 40s)

Similarly, another participant shared the lack of awareness about the effects of betel nuts on cardiovascular health which warrants focused education:

I eat chhalia but I do not believe it has any relationship with the heart disease… (P16, F, early 40s)

### Theme 3: holistic perspectives and health management

Holistic perspectives on health management involve a diverse approach that integrates various aspects of an individual’s health. Participants with coronary angioplasty exhibit a proactive approach to their cardiovascular health, emphasising the importance of preventive measures and self-management strategies. This proactive stance, evident in their commitment to remaining active and avoiding potential triggers for another heart attack, signifies a comprehensive approach beyond medical interventions.

Additionally, there is an awareness of the association between physical inactivity and the risk of heart disease, underlining the significance of an active lifestyle in alleviating heart-related risks. Participants further acknowledge the multifactorial causes of heart disease, considering factors such as family history, obesity, smoking and diabetes. This recognition extends to a divine perspective, suggesting a holistic understanding encompassing both natural and spiritual dimensions. The awareness of blood pressure and sugar levels adds another layer to health management, highlighting the significance of monitoring key health indicators. Finally, the evidence of positive behavioural change postheart attack reflects a commitment to adopting healthier habits, completing the holistic approach by addressing physical aspects and behavioural and lifestyle factors to pursue overall health.

#### Subtheme 3.1: proactive approach with coronary angioplasty

Patients with coronary angioplasty express a proactive approach to health, aiming to remain active and avoid potential triggers for another heart attack. This proactive mindset demonstrates a commitment to preventive measures and overall health. The patients’ responses reflect a reflective awareness and a sense of urgency regarding the gravity of their health condition, particularly cardiovascular issues. The statement suggests a critical moment of realisation, perhaps triggered by the experience of having undergone 2–3 heart stent procedures. The patient seems to believe that this health event is a pivotal point in their life, emphasising a conviction that neglecting self-care could lead to dire consequences.

Furthermore, the mention of having ‘no chance for the next’ implies a perceived vulnerability and a risk associated with potential subsequent cardiac events. The patient’s proactive approach to self-care is evident in their commitment to remaining active and avoiding factors that may contribute to another heart attack. Overall, the significance of personal responsibility and lifestyle choices in managing and preventing cardiovascular issues, offering valuable insights into the patient’s mindset and motivation for adopting healthier habits can be witnessed in the participant’s response:

I thought at that time that it is first and last if I do not care for myself this time…because if I get the second, I will be over…I had 2–3 coronary angioplasty in my heart…there is no chance for the next…so I teach myself to remain active and avoid things that cause heart attack… (P20, M, mid 50s)

#### Subtheme 3.2: inactivity and heart disease

The responses from patients, specifically the statement *I think those who remain inactive get heart disease,* carry a belief or perception regarding the relationship between physical inactivity and the development of heart disease. It suggests an awareness among the patients of the potential health risks associated with a sedentary lifestyle.

From an interpretative viewpoint, this response highlights the importance of health education and awareness campaigns, as participants seem to recognise the connection between physical activity and cardiovascular health. It may also reflect a level of health literacy among the respondents. In a healthcare context, this insight could be valuable for healthcare providers and public health initiatives aiming to promote preventive measures against heart disease. Moreover, it could serve as a starting point for interventions encouraging physical activity and healthy lifestyle choices, as participants already acknowledge the potential consequences of remaining inactive. Ultimately, understanding and addressing such perceptions can contribute to more targeted and effective health promotion strategies:

I think those who remain inactive get heart disease… (P18, M, late 50s)

#### Subtheme 3.3: disease as divine perspective

On the other hand, the patient also brings a spiritual perspective into the conversation, attributing the ultimate cause to a divine power, expressing a sense of acceptance and resignation by stating*, It is from almighty ALLAH.* This response suggests an understanding of health encompassing genetic factors and a belief in divine influence, highlighting the complex interplay of biological and spiritual explanations for illness. It also emphasises the importance of cultural and personal beliefs in shaping patients’ perceptions of health and disease.

I think heart disease runs in the family…my mother and father have this disease, so I have…people kept on searching for the cause, but they need to understand that it is from almighty ALLAH… (P5, M, late 60s)

Another participant shared a similar understanding about the disease’s connection with divine power while at the same time the response reflected a lack of awareness about the causes:

I have no idea what is the reason for my disease…I believe it is from Almighty… (P19, F, Early 50s)

#### Subtheme 3.4: awareness of blood pressure and sugar

The finding reveals a patient’s acknowledgement of neglecting their hypertension, attributing it to a lack of seriousness in lifestyle choices such as smoking and consuming fatty foods. The realisation that smoking may have played a role in the development of the disease indicates an emerging awareness of the link between lifestyle factors and health conditions. This response suggests a shift in attitude, with the patient recognising the need for a more proactive approach to their health, possibly reflecting a motivation for behaviour change:

I had hypertension, and I did not take it seriously…I kept on smoking and used to eat fatty foods…I realize that smoking has caused me this disease… (P4, M, early 70s)

Furthermore, the response provides a detailed account of a patient’s postheart attack experience, emphasising the challenges of managing blood pressure and sugar levels. The patient’s admission to taking extra pills before the heart attack suggests a history of non-adherence to prescribed medications, possibly contributing to the exacerbated condition. The detailed medication regimen indicates a more structured approach to managing hypertension postheart attack. The mention of sugar disturbance highlights the interconnectedness of health issues and the need for a holistic approach to managing multiple conditions:

Before my heart attack, I used to take extra pills for my blood pressure, which used to increase frequently. I take two different blood pressure medications now: a 10 mg one that I take twice daily and a 5 mg one that I take only once. It is not only BP, but the sugar also disturbed me a lot…I do not take too much sugar but a very little occasionally. (P6, M, early 70)

The participant shared the importance of awareness and knowledge in managing heart-related conditions. The patient advocates for heart patients to be informed about normal blood pressure and sugar levels, suggesting that awareness empowers patients to manage their health proactively. The statement serves as a call to action, emphasising the consequences of neglecting these parameters and the potential for self-inflicted health issues. This response suggests a belief in the role of education and self-awareness in preventing complications and maintaining cardiovascular health:

Heart patients need to know what normal BP and sugar are because if they know they can manage it easily and if they ignore, they invite trouble for themselves. (P11, M, early 50s)

#### Subtheme 3.5: behavioural change

The response from the patient indicates an awareness and acknowledgement of a previous detrimental lifestyle or health-related behaviour. The statement *I realized that my behavior towards my health was not good at that time* reflects a moment of self-awareness, suggesting that the individual recognised and admitted to engaging in habits or actions that were detrimental to their health. The subsequent comment, *But now I have changed it significantly,* conveys a positive transformation of patient behaviour. This shift implies that the patients have consciously tried to modify their habits or adopt healthier practices. The word ‘*significantly’* suggests a substantial and meaningful change, emphasising the importance of behaviour change. This qualitative response indicates a positive health behaviour intervention or lifestyle modification, and it stresses the potential for patients to reflect on and take proactive steps towards improving their health:

I realized that my behavior was not good at that time towards my health, but now I have changed it significantly… (P8, M, early 50s)

## Discussion

The findings from the present study align with and complement existing literature on the multifaceted nature of risk factors contributing to heart disease. The participants in this study demonstrated an understanding of various elements influencing cardiovascular health, which resonates with similar themes found in the recent literature. The majority of participants demonstrated an understanding of risk factors after the development of heart disease, while others still overlooked the independent risk factors of CAD.

Recognising unhealthy dietary habits as a significant risk factor for heart disease corresponds with research emphasising the impact of high-fat and meat-rich diets on cardiovascular health.^5^ Due to the cultural practice of consuming more rice, wheat and refined carbohydrates, the risk of heart disease still dominates in Pakistani culture. Additionally, the awareness of the link between nutrition and heart-related risks underscores the importance of promoting healthy dietary choices for preventing heart disease.^6^ However, limited financial resources and the capacity to spend on themselves hamper them from eating more protein and fibre diet. Furthermore, the participants’ concern about cholesterol levels and their association with dietary choices aligns with studies emphasising the role of cholesterol in cardiovascular diseases.^7^ The concerns about cholesterol in patients remain a significant worry factor because of the nature of work and society’s dietary patterns, including ghee, butter and deep-fried food. The participants’ emphasis on managing individual health markers demonstrates a proactive approach towards cardiovascular health.^8^

The recent realisation among participants regarding the adverse effects of smoking on heart health corresponds with recent literature highlighting the harmful impact of smoking on cardiovascular health.^9^ Moreover, the critical reflection on past habits indicates a rising awareness of the need for smoking cessation as a preventive measure against heart disease.^10^ Additionally, acknowledging the combined impact of smoking and diabetes on heart disease aligns with studies emphasising the synergistic effects of multiple risk factors. Recognising these connections reflects a deeper understanding of the complex interplay of various factors contributing to cardiovascular health.^11^ The findings also showed a careless attitude towards their health with a smoking habit, which patients did for pleasure. Due to habitual smoking, many of them face trouble stopping it.

The retrospective evaluation of past lifestyles and the acknowledgement of excessive smoking as a contributor to heart conditions resonate with the literature emphasising the importance of recognising and addressing past behaviours for better health.^12^ Furthermore, participants’ positive lifestyle changes, including smoking cessation, are consistent with interventions promoting healthier lifestyles for patients at risk of heart disease.^13^ The reported improvement in health supports the literature advocating for lifestyle modifications as effective preventive measures.^5^ The findings demonstrate a need for designing a structured programme using non-pharmacological behavioural interventions for patients who are willing to stop smoking after experiencing a heart attack

The acknowledgement of the role of substances like chhalia, smokeless tobacco and caffeine in contributing to heart disease aligns with recent studies highlighting the diverse risk factors beyond conventional ones.^14^ This broader recognition suggests a more comprehensive understanding of the factors influencing cardiovascular health. Additionally, the proactive approach expressed by patients with coronary angioplasty aligns with the literature emphasising the importance of preventive measures and self-management strategies for patients with cardiovascular issues.^15^ This attitude reflects a commitment to overall health and minimising the risk of future heart attacks, which can be achieved by offering them a feasible rehabilitation programme for self-management following a heart attack.

The awareness of the association between physical inactivity and the risk of heart disease corresponds with the existing literature emphasising the importance of regular physical activity as a preventive measure for cardiovascular health.^16^ Recognising the importance of remaining active is crucial for modifying heart-related risk behaviours. Furthermore, the acknowledgement of heart disease as a result of a combination of factors, including family history, obesity, smoking and diabetes, aligns with the multifactorial nature of heart disease causation discussed in contemporary research.^17^ This recognition highlights the complexity of cardiovascular risk factors. However, patients must be counselled for this complex relationship to prevent future cardiovascular events.

The attribution of heart disease to family history, coupled with a divine perspective, reflects a holistic understanding of health that encompasses both natural and spiritual dimensions. This perspective resonates with the literature emphasising the need for a comprehensive and culturally sensitive approach to heart health.^8^ Linking a disease with divine power despite possessing poor healthy behaviours depicts a poor understanding of definite causes of heart diseases, which need to be improved after the initial treatment during a hospital stay.

The emphasis on managing blood pressure and sugar levels aligns with the existing literature stressing the significance of controlling these vital health markers for preventing heart-related complications.^18^ This awareness highlights the importance of regularly monitoring and managing key health indicators.

The evidence of positive behavioural change postheart attack corresponds with studies showcasing the effectiveness of interventions to promote healthier habits among patients with a history of heart disease.^19^ Furthermore, the participant’s commitment to adopting healthier behaviours reflects a proactive stance towards improving heart health outcomes. Despite the non-availability of structured rehabilitation programmes, patients are still helping themselves to make significant changes in their lifestyle, which is not enough. There needs to be a structured programme based on the Pakistani context, so that patients can get maximum benefit from it.

Moreover, participants demonstrate an understanding of the synergistic effects of smoking and diabetes on heart disease. The data show a cognitive shift towards recognising past behaviours that may have contributed to their heart condition, with several participants expressing positive lifestyle changes, including quitting smoking. Beyond conventional risk factors, participants acknowledge the role of substances like chhalia, smokeless tobacco and caffeine in contributing to heart disease, reflecting a broader recognition of diverse risk factors. The research further highlights a proactive mindset among patients with coronary angioplasty in their hearts, indicating a commitment to preventive measures and overall health.

### Recommendations

Based on the results obtained from the participants’ responses, several research recommendations can be proposed to explore further and understand the complex relationship of factors influencing heart disease:

1. Conduct longitudinal studies to track patients who have undergone positive lifestyle changes postheart attack. Investigate the sustainability of these changes and their impact on cardiovascular health over an extended period.
2. Investigate the influence of cultural and spiritual beliefs on patients’ perceptions of health, particularly regarding heart disease. Explore how incorporating cultural and spiritual dimensions into health interventions may enhance effectiveness.
3. Design, implement and evaluate home-based interventions using patients’ reported experiences that prevent heart disease through education, lifestyle modification and early detection.
4. Research and develop innovative approaches for promoting positive behavioural changes postheart attack. Explore using technology, social networks and community engagement to facilitate sustained behaviour change.
5. Evaluate the effectiveness of interventions within the healthcare system, such as patient education, counselling and support services, in promoting healthy behaviours and quality of life and preventing the recurrence of heart attacks.

These research recommendations aim to build on the understanding revealed by participants and contribute to developing targeted interventions such as Home-based Cardiac Rehabilitation (HBCR) for preventing and managing heart disease.

### Conclusion

Participants recognise the connection between physical inactivity and the risk of heart disease, emphasising the importance of remaining active. The acknowledgement of a combination of factors, including family history, obesity, smoking and diabetes, highlights an understanding of the multifactorial nature of heart disease causation. Some participants bring a holistic perspective, attributing certain aspects to a divine perspective, reflecting an understanding of health that encompasses both natural and spiritual dimensions.

Additionally, recognising that managing blood pressure and sugar levels is vital to preventing heart-related complications showcases participants’ understanding of these health markers. The evidence of positive behavioural change postheart attack suggests a commitment to adopting healthier habits, with participants realising past unhealthy behaviours and actively implementing changes for better heart health. The findings contribute rich qualitative data to inform interventions and strategies for promoting heart health awareness and preventive measures in diverse populations.

### Patient and public involvement

Before starting the final interviews with the research participants, the pilot qualitative interviews were conducted with heart patients to see the clarity and understandability of questions by patients. Patients included in the pilot interviews were excluded from the primary sample size. After the pilot interviews, a few questions were modified based on the responses received, and final interviews were conducted until data saturation.

## Data Availability

Data are available upon reasonable request.
