# Supplementary material for: Qualitative exploration of awareness about risk factors and transformative behaviours among Pakistani patients with coronary artery disease
Source: BMJ Open. 2024 Nov 24;14(11):e084566. doi: 10.1136/bmjopen-2024-084566 (PMC11590824; doi:10.1136/bmjopen-2024-084566)
Supplement: Supplementary file 1 [file bmjopen-2024-084566.reviewer_comments.pdf]

PEER REVIEW HISTORY

BMJ Open publishes all reviews undertaken for accepted manuscripts. Reviewers are asked to complete a checklist review form and are provided with free text boxes to elaborate on their assessment. These free text comments are reproduced below.

ARTICLE DETAILS

Title (Provisional)

Qualitative Exploration of Awareness about Risk Factors and Transformative Behaviors among Pakistani Patients with Coronary Artery Disease

Authors

Barolia, Rubina; YAQOOB, Adnan; Ladak, Laila; Khan, Aamir Hameed; Pannu, Furqan Yaqub

---

VERSION 1 - REVIEW

**Reviewer**	**1**
**Name**	**Riaz , Muhammad Shahid**
**Affiliation**	**The University of Lahore, Lahore School of Nursing**
**Date**	**14-Mar-2024**
**COI**	**None**

The has reported significant contextual experiences/journeys of Pakistani patients after suffering from a heart attack. The findings reported in this paper would help the researcher understand patients' perceptions and challenges in making lifestyle changes at the same time would help the researcher develop future interventions for cardiac patients to speed up their recovery process.

However, the authors need to mention the qualitative research questions under separate heading after the introduction and abstract as well.

**Reviewer**	**2**
**Name**	**Sabghatullah, Hafiz**
**Affiliation**	**Bahria Town School of Nursing , Principal's Office**
**Date**	**14-Mar-2024**
**COI**	**No competing interest to this research article.**

Suggestion for the author

1. It would be best if aims and objectives are clearly mentioned in the study

2. it

**Reviewer**	**3**
**Name**	**Naz, Anila**
**Affiliation**	**University of Alberta, Faculty of Nursing**
**Date**	**15-Mar-2024**
**COI**	**NO COMPETING INTERESTS**

Overall, it is a very well-written qualitative research paper, and the results presented are insightful and contribute significantly to the field. Just a suggestion: There is some redundant information in the recommendation section. So, the authors can remove either point 2 or point 4.

**Reviewer**	**4**
**Name**	**Kokab, Farina**
**Affiliation**	**University of Birmingham**
**Date**	**07-May-2024**
**COI**	**N/A**

Thank you for undertaking this work and writing a clear and concise paper on the implications of health beliefs on preventing heart disease.

**Reviewer**	**5**
**Name**	**Armstrong, David**
**Affiliation**	**King's College London**
**Date**	**15-Aug-2024**
**COI**	**none**

This paper describes the knowledge of cardiovascular risk factors in a Pakistani population post-MI.

Risk factor epidemiology first emerged about 60 years ago. A risk factor was not a definitive ‘cause’ of any disease but rather increased the probability of it emerging. For some risk factors, such as smoking, the probability was quite high but nevertheless most patients who smoked did not get heart attacks. Other risk factors have even smaller effect sizes and the effect of some of them is still debated. This paper however takes a fairly absolutist view of

risk factors reporting them as more causal factors rather than risk factors. Some of the risk factors described here as being implicated in cardiovascular disease such as coffee, red meat and betel nuts are either debated in the medical literature or, if they do have an effect, it is very small. This is not to deny that the presence of multiple risk factors increases the probability of heart disease, but it is far from certain that an MI will occur.

The study involved interviews with 20 patients about six months after their MI and asked them about their knowledge of risk factors. These interviews were transcribed and analysed qualitatively. For most of the risk factors mentioned by the patients, the authors confirmed their validity by referring to the medical literature – and for the main part the patients were 'accurate'. But surely the patients get it 'right' because they are simply repeating what they have been told. All these patients had an MI six months ago and have been under medical care since and no doubt have had lots of advice from doctors on why this happened and how they can prevent recurrence. Patients don't naturally worry about their cholesterol levels without being told about them by their doctors. So, in effect this is not a study of patients' knowledge of risk factors but rather patients' ability to repeat what they have recently been told by their doctors. The more appropriate question for this study therefore is not 'patients' understanding of the perceived risk factors of heart attack and their lifestyle changes' but can they recall what they have been told and what sense do they make of that information. But that would have required a different interview schedule. Besides, there is evidence that patients are quite good at repeating what they have been told while their adherence to the medical message can be less than ideal.

About 30 years ago Davison et al (https://doi.org/10.1111/j.1467-9566.1991.tb00085.x) published an influential paper on 'lay epidemiology and the prevention paradox' in which they interviewed patients about their knowledge of risk factors and found a range of views, many factors, such as having a red face, not being recognised by medical research (unlike the views expressed in this paper that closely follow medical opinion). Davison et al also picked up on Rose's prevention paradox in that change in risk factor behaviour can benefit a population but have minimal effect on individuals. As this paper purports to explore lay views of risk factors – a sort of lay epidemiology – it would have been much more appropriate to refer to this more anthropological literature rather than immersing the study in medical research. But the major problem, as indicated, is that the study does not in fact investigate lay understanding of risk factors but rather whether patients can recall what they have been told by their medical attendants.

VERSION 1 - AUTHOR RESPONSE

Reviewers' Comments	Response to Reviewers' Comments	Page #
**Reviewer: 1**<br>Dr. Muhammad Shahid Riaz, The University of Lahore<br>Comments to the Author:<br>The has reported significant contextual experiences/journeys of Pakistani patients after suffering from a heart attack. The findings reported in this paper would help the researcher understand patients' perceptions and challenges in making lifestyle changes at the same time would help the researcher develop future interventions for cardiac patients to speed up their recovery process.<br>However, the authors need to mention the qualitative research questions under separate heading after the introduction and abstract as well.	Thank you for feedback.<br><br>qualitative research questions are mentioned under separate heading and highlighted	2
**Reviewer: 2**<br>Dr. Hafiz Sabghatullah, Bahria Town School of Nursing<br>Comments to the Author:<br>Suggestion for the author<br><br>1. It would be best if aims and objectives are clearly mentioned in the study<br>2. it	Thank you for feedback.<br><br>Objectives are mentioned and highlighted	2
**Reviewer: 3**<br>Dr. Anila Naz, University of Alberta<br>Comments to the Author:<br>Overall, it is a very well-written qualitative research paper, and the results presented are insightful and contribute significantly to the field. Just a suggestion: There is some redundant information in the recommendation section. So, the authors can remove either point 2 or point 4.	Thank you for feedback.<br><br>Redundancy removed, recommendation section highlight	22
**Reviewer: 4**<br>Dr. Farina Kokab, University of Birmingham<br>Comments to the Author:<br>Thank you for undertaking this work and writing a clear and concise paper on the implications of health beliefs on preventing heart disease.	Thank you for feedback.	-
**Reviewer: 5**<br>Dr. David Armstrong, King's College London<br>Comments to the Author:<br>This paper describes the knowledge of cardiovascular risk factors in a Pakistani population post-MI.	Thank you very much for your thoughtful and detailed feedback on our manuscript. We appreciate the opportunity to clarify our position and address the important points you raised.	-
Risk factor epidemiology first emerged about 60 years ago. A risk factor was not a definitive ‘cause’ of any disease but rather increased the probability of it emerging. For some risk factors, such as smoking, the probability was quite high	We fully agree with your assessment that risk factors should be viewed as probabilistic contributors rather than definitive causes of disease. Our intention was not to suggest an	-

but nevertheless most patients who smoked did not get heart attacks. Other risk factors have even smaller effect sizes and the effect of some of them is still debated. This paper however takes a fairly absolutist view of risk factors reporting them as more causal factors rather than risk factors. Some of the risk factors described here as being implicated in cardiovascular disease such as coffee, red meat and betel nuts are either debated in the medical literature or, if they do have an effect, it is very small. This is not to deny that the presence of multiple risk factors increases the probability of heart disease, but it is far from certain that an MI will occur.	absolutist or deterministic view but to emphasize the perceived significance of certain risk factors in the context of our study population. In Pakistan, there are cultural and behavioral differences that may heighten the public's focus on specific risk factors, such as betel nut chewing, which has a high prevalence in the region and has been associated with various cardiovascular and health risks.<br><br>While we acknowledge that the effect sizes of factors like coffee, red meat, and betel nuts are debated in the broader medical literature, our study focused on the patients' perceptions of these factors. These perceptions are shaped not only by medical advice but also by cultural beliefs and societal practices. Thus, our goal was to document these perceptions as they were expressed by the patients, reflecting their perceived risk rather than asserting these factors as conclusive causes of cardiovascular disease.	
The study involved interviews with 20 patients about six months after their MI and asked them about their knowledge of risk factors. These interviews were transcribed and analysed qualitatively. For most of the risk factors mentioned by the patients, the authors confirmed their validity by referring to the medical literature – and for the main part the patients were 'accurate'. But surely the patients get it 'right' because they are simply repeating what they have been told. All these patients had an MI six months ago and have been under medical care since and no doubt have had lots of advice from doctors on why this happened and how they can prevent recurrence. Patients don't naturally worry about their cholesterol levels without being told about them by their doctors. So, in effect this is not a study of patients' knowledge of risk factors but rather patients' ability to repeat what they have recently been told by their doctors. The more appropriate question for this study therefore is not 'patients' understanding of the perceived risk factors of heart attack and their lifestyle changes' but can they recall what they have been told and what sense do they make of that information. But that would have required a different interview schedule. Besides, there is evidence that patients are quite good at repeating	You raise an excellent point regarding the possibility that patients may be reflecting medical advice rather than an independent understanding of risk factors. Our objective was to explore patients' perceptions while it is true that their responses may reflect recent medical guidance. We aimed to understand how patients perceived their risk factors and how they translated this understanding into transformative behaviors post-MI. It is also crucial to recognize that the way patients act on this information is central to managing their long-term health outcomes. Even if much of their knowledge came from medical advice, the study sheds light on how patients conceptualize and personalize these risk factors, which is critical for their adherence to long-term health interventions.<br>We agree that pre-MI knowledge of risk factors would offer a different perspective, but given the nature of the study and the timing of the interviews, we sought to explore how recent experiences, including their	-

what they have been told while their adherence to the medical message can be less than ideal.	interactions with healthcare providers, shaped their risk perceptions and behaviors. The study highlights the importance of patient education in reinforcing these perceptions and motivating lifestyle changes.<br>We have acknowledged in the limitations of this study that captures post-event perceptions influenced by medical advice which can be a recall bias. (*Strengths and Limitations*)	
About 30 years ago Davison et al (https://doi.org/10.1111/j.1467-9566.1991.tb00085.x) published an influential paper on 'lay epidemiology and the prevention paradox' in which they interviewed patients about their knowledge of risk factors and found a range of views, many factors, such as having a red face, not being recognised by medical research (unlike the views expressed in this paper that closely follow medical opinion). Davison et al also picked up on Rose's prevention paradox in that change in risk factor behaviour can benefit a population but have minimal effect on individuals. As this paper purports to explore lay views of risk factors – a sort of lay epidemiology – it would have been much more appropriate to refer to this more anthropological literature rather than immersing the study in medical research. But the major problem, as indicated, is that the study does not in fact investigate lay understanding of risk factors but rather whether patients can recall what they have been told by their medical attendants.	Thank you for pointing us to the work of Davison et al. and the concept of lay epidemiology. While we agree that this literature provides a valuable anthropological perspective, our study was specifically designed to explore how patients' understanding of risk factors aligns with the medical framework, particularly in a resource-limited setting like Pakistan. The study was focused on how patients interpret and act upon medical advice, rather than uncovering a broader, lay epidemiology approach.<br><br>Once again, we thank you for your constructive feedback. Your comments have allowed us to deepen our team's discussion and internalization of thoughts. We hope that our clarifications address your concerns.	-

VERSION 2 - REVIEW

**Reviewer**	**5**
**Name**	**Armstrong, David**
**Affiliation**	**King's College London**
**Date**	**20-Oct-2024**
**COI**	

There are two ways of framing this paper. The first is as an investigation of patient perceptions of risk factors for heart disease. Perceptions here would refer to what patients think, whether it is (medically) right or wrong. The title and some of the text still implies this is the approach being adopted. But, on reading this revision I realise that the research

question is not about the patient's world-view but rather about their awareness of medically proven risk factors. I have approached my review with this latter question in mind.

If indeed the paper is concerned with patient awareness of risk factors (medically defined) then some of the language around perception could be revised. For example, the paper is not 'exploring participants' experiences and attitudes' (though some of these are mentioned as existing in the past) but instead 'seeks to the depth of awareness regarding the connection between lifestyle and cardiovascular health'. And the first research question, 'What are the perceptions and concerns of Pakistani patients about the risk factors of CAD' could be reworded to cover that it is awareness of risk factors that is being addressed in the paper.

The data collection methods section should be amended to include the background of the interviewer/researcher. The interviews were conducted either in the outpatient department or by telephone, but it may matter whether the interviewer was seen as part of the medical team or someone independent. The former may have biassed results with patients anxious to please and say the right things. If so, this needs to be covered in a section on study limitations later in the paper. Also in this section, the statement that 'Data saturation in this study was achieved when no new information was received from the participants' needs revising. Data saturation refers to no additional themes (or equivalent) emerging not 'information'.

Table 1 offers an overview of the findings included in the results with the broad themes listed together with representative quotes. I counted 23 quotes, 22 from male participants and 1 from a female. It would be nice to have a more balanced representation as surely the 6 female out of 20 participants said something worthwhile in interviews lasting 40-50 minutes? Further, two male participants get into the table twice with exactly the same quote though listed under different themes. I suppose it is possible for a quote to support two different themes but it does hint at under-analysis and it would be helpful to remove this duplication.

The results explore patients' awareness of cardiovascular risk factors and as far as I can see all the participants seemed to be fully aware of them – the only exception was the participant who thought 'very little' smoking was OK. Otherwise, I did not see any examples of where patients reported lack of risk factor awareness. This finding, or implication, that all participants seem to get it right, does rather undermine the conclusion that patient education is important for these patients: if they already know the risk factors and act on them, as reported here, what is the value of further education? Either there should be some 'ignorance' reported or the conclusion changed.

With these specific revisions I think the paper would be more suitable for publication.

VERSION 2 - AUTHOR RESPONSE

S.No	Reviewers' Comments	Response to Reviewers' Comments	Page #
	**Previous Title of Study**<br>"Qualitative Exploration of Perceived Risk Factors and Transformative Behaviors among Pakistani Patients with Heart Disease"<br>As the respected reviewer's comments, the paper will be reworded around awareness rather than perception. Therefore the title has been revised accordingly.	**Updated Title of Study**<br>"Qualitative Exploration of Awareness about Risk Factors and Transformative Behaviors among Pakistani Patients with Heart Disease"	1
	Please revise points 2 and 4 in the 'Strengths and limitations' section of your manuscript (after the abstract). This section should contain up to five short bullet points, no longer than one sentence each, that relate specifically to the methods. The novelty, aims, results or expected impact of the study should not be summarized here.	Thank you,<br>Points 2 and point 4 have been modified and highlighted.	1
	Along with your revised manuscript, please include a copy of the SRQR checklist for reporting qualitative research, indicating the page/line numbers of your manuscript where the relevant information can be found (http://journals.lww.com/academicmedicine/fulltext/2014/09000/Standards_for_Reporting_Qualitative_Research___A.21.aspx)	Revised and uploaded	
	Please name the ethics committee that approved your study in the Methods section.	Thank you for the suggestion.<br>The ethics committee name has been added and highlighted	5
	There are two ways of framing this paper. The first is as an investigation of patient perceptions of risk factors for heart disease. Perceptions here would refer to what patients think, whether it is (medically) right or wrong. The title and some of the text still implies this is the approach being adopted. But, on reading this revision I realise that the research question is not about the patient's world-view but rather about their awareness of medically proven risk factors. I have approached my review with this latter question in mind.	Thank you for showing us the right direction. The paper has been rewarded to follow the concept of awareness rather than perception.	-

	If indeed the paper is concerned with patient awareness of risk factors (medically defined) then some of the language around perception could be revised. For example, the paper is not 'exploring participants' experiences and attitudes' (though some of these are mentioned as existing in the past) but instead 'seeks to the depth of awareness regarding the connection between lifestyle and cardiovascular health'. And the first research question, 'What are the perceptions and concerns of Pakistani patients about the risk factors of CAD' could be reworded to cover that it is awareness of risk factors that is being addressed in the paper.	The first question and objective no. 1 have been updated and highlighted.<br><br>**Research Questions:**<br>What is the awareness of Pakistani patients about the risk factors of CAD?<br>**Research Objectives:**<br>To explore Pakistani patients' awareness of risk factors for CAD.	2
	The data collection methods section should be amended to include the background of the interviewer/researcher. The interviews were conducted either in the outpatient department or by telephone, but it may matter whether the interviewer was seen as part of the medical team or someone independent. The former may have biased results with patients anxious to please and say the right things. If so, this needs to be covered in a section on study limitations later in the paper.	The research team conducted interviews with participants once they consented. The Research team was not part of the medical team who were involved in treating the patients.	-
	Also in this section, the statement that 'Data saturation in this study was achieved when no new information was received from the participants' needs revised. Data saturation refers to no additional themes (or equivalent) emerging not 'information'.	Thank you for the correction. Corrected and highlighted.<br>Data saturation in this study was achieved when no new themes emerged from the participants' data.	4
	Table 1 offers an overview of the findings included in the results with the broad themes listed together with representative quotes. I counted 23 quotes, 22 from male participants and 1 from a female. It would be nice to have a more balanced representation as surely the 6 female out of 20 participants said something worthwhile in interviews lasting 40-50 minutes?	Quotes from other female participants were included in the results and table. Thank you for this suggestion. Please see highlighted changes on page numbers 8,11,16,19	8,11,16,19
	Further, two male participants get into the table twice with the same quote though listed under different themes. I suppose it is possible for a quote to support two different themes but it does hint at under-	Thank you for mentioning, the redundancy was removed from the Table and text.	5-7

	analysis and it would be helpful to remove this duplication.		
	The results explore patients' awareness of cardiovascular risk factors and as far as I can see all the participants seemed to be fully aware of them – the only exception was the participant who thought 'very little' smoking was OK. Otherwise, I did not see any examples of where patients reported a lack of risk factor awareness. This finding, or implication, that all participants seem to get it right, does rather undermine the conclusion that patient education is important for these patients: if they already know the risk factors and act on them, as reported here, what is the value of further education? Either there should be some 'ignorance' reported or the conclusion changed.	Thank you for your comment. Some participants in this group were unaware of the risk factors of heart disease which I have now included in the results. However, others have now realized that their initial understanding of the risk factors was not correct. I want to mention here that these patients were those who suffered a heart attack and had a minimum of 6 months of experience dealing with it. However, those in the early phase of recovery still have a few misconceptions which need to be resolved earlier to speed up their recovery. therefore, education is needed for those who recently suffered a heart attack so that early modification in behavior could lead to swift recovery.	-

VERSION 3 - REVIEW

**Reviewer**	**5**
**Name**	**Armstrong, David**
**Affiliation**	**King's College London**
**Date**	**29-Oct-2024**
**COI**	

Can I thank the authors for attending to all the points I raised in my earlier review. I think the paper is now suitable for publication.
